# Global network community and non-uniform cell density in the macaque brain

**DOI:** 10.1186/1471-2202-15-S1-P100

**Published:** 2014-07-21

**Authors:** Masanori Shimono

**Affiliations:** 1 Department of Physics, University of Indiana, Bloomington, IN, 47405, USA

## 

The important question, how the global network architecture connecting cortical regions keeps balances between integration and segregation of information processes, have been asked to understand the design of the brain [1,2]. This study aimed to clarify how topological characteristics of such global network architecture relate with physiological characteristics inside of segmented cortical regions in the monkey brain [3]. Especially, I focused on cell densities (densities of neurons or non-neurons) as the representative characteristics of segmented cortical regions [4], and compared the cell densities with network topologies of cortico-cortical fiber tracts [Figure1-A]. To reduce biases in comparisons, I surveyed many topological measures as wide as possible. Total number of evaluated network measures was 27.

As the result, surprisingly, only participation coefficient (PCs) showed significant correlations with cell densities [3]. Although a previous study reported that cell densities significantly change on the anterior-posterior coordinate [5], spatial coordinates did not correlate significantly with participation coefficients. Participation coefficient is the topological measure evaluating how often each node (segmented brain region) connects to other nodes locating different communities (modules). The modules, which detected based on a computational criterion [3], corresponded with visual, somatosensory, auditory, and two associative modules [Figure1-C]. The associative modules simultaneously showed low neuron density and high participation coefficient, which means there are diversive connections with different modules. These findings led us to the conclusion that the brain is designed for achieving integrative information process at associative brain regions by sacrificing number of elements (neurons).

**Figure1:**
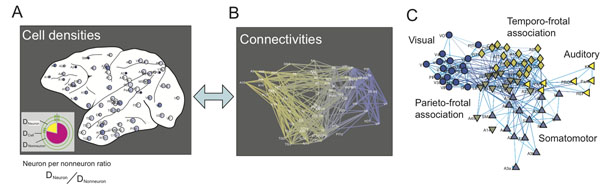
(A) is cell densities at segmented 69 cortical regions. Denser color at each region indicates higher density of neurons. (B) is the network organization of white matter fibers. (C) Community (Modular) structures of monkey cortical networks. The locations of nodes were determined using the Fruchterman–Reingold algorithm. Differences of markers indicate five different modules determined using a community detection algorithm.

## References

[B1] Chen Y Wang S Hilgetag CC Zhou C Trade-off between Multiple Constraints Enables Simultaneous Formation of Modules and Hubs in Neural PLoS computational biology 2013 15 3 e1002937 10.1371/journal.pcbi.1002937 23505352 PMC3591279

[B2] Sporns O Network attributes for segregation and integration in the human brain Current opinion in neurobiology 2013 23294553 10.1016/j.conb.2012.11.015

[B3] Shimono M Non-uniformity of cell density and networks in the monkey brain Scientific reports 2013 15 23985926 10.1038/srep02541 PMC3756338

[B4] Collins CE Airey DC Young NA Leitch DB Kaas JH Neuron densities vary across and within cortical areas in primates Proc Natl Acad Sci USA 2010 15 36 15927 15932 10.1073/pnas.1010356107 20798050 PMC2936588

[B5] Cahalane DJ Charvet CJ Finlay BL Systematic, balancing gradients in neuron density and number across the primate isocortex Frontiers in Neuroanatomy 2012 15 28 22826696 10.3389/fnana.2012.00028 PMC3399120

